# Cysticercosis-related Deaths, California

**DOI:** 10.3201/eid1003.020749

**Published:** 2004-03

**Authors:** Frank J. Sorvillo, Lawrence Portigal, Christopher. DeGiorgio, Lisa Smith, Stephen H. Waterman, George W. Berlin, Lawrence R. Ash

**Affiliations:** * UCLA School of Public Health, Los Angeles, California, USA; † Office of Vital Records, State of California, Sacramento, California, USA; ‡ UCLA School of Medicine, Los Angeles, California, USA; § Centers for Disease Control and Prevention, Atlanta, Georgia, USA

**Keywords:** cysticercosis, mortality, epidemiology, population surveillance, public health

## Abstract

Cysticercosis is an increasingly important disease in the United States, but information on the occurrence of related deaths is limited. We examined data from California death certificates for the 12-year period 1989–2000. A total of 124 cysticercosis deaths were identified, representing a crude 12-year death rate of 3.9 per million population (95% confidence interval [CI] 3.2 to 4.6). Eighty-two (66%) of the case-patients were male; 42 (34%) were female. The median age at death was 34.5 years (range 7–81 years). Most patients (107, 86.3%) were foreign-born, and 90 (72.6%) had emigrated from Mexico. Seventeen (13.7%) deaths occurred in U.S.-born residents. Cysticercosis death rates were higher in Latino residents of California (13.0/10^6^) than in other racial/ethnic groups (0.4/10^6^), in males (5.2/10^6^) than in females (2.7/10^6^), and in persons >14 years of age (5.0/10^6^). Cysticercosis is a preventable cause of premature death, particularly among young Latino persons in California and may be a more common cause of death in the United States than previously recognized.

Cysticercosis, an infection caused by the larval form of the pork tapeworm, *Taenia solium*, is recognized as an increasingly important cause of severe neurologic disease in the United States (*1*–*3*). In the typical transmission cycle, eggs from the adult tapeworm are shed in the feces of a human carrier and subsequently ingested by pigs, the usual intermediate host (*4*). Larvae emerge from the eggs, penetrate the intestinal mucosa and disseminate through the bloodstream to various tissues where the larval stage or cysticercus develops. The cycle is completed when humans, the only naturally infected definitive host, consume raw or undercooked pork containing cysticerci, which attach to the small bowel and develop into adult tapeworms, thereby completing the cycle. However, humans may also become infected with the larval stage when eggs, which are directly infectious, are ingested, typically through contaminated food or water. Neurocysticercosis, the most severe form of the disease, occurs when larvae invade tissue of the central nervous system. While cysticercosis in the United States principally affects immigrants from cysticercosis-endemic areas of Latin America, it has been increasingly recognized in U.S.-born residents as well (*5*–*7*).

Despite the growing importance of cysticercosis, surveillance systems for cysticercosis have rarely been implemented (*6*,*8*), and the true impact of the disease in the United States is largely unknown. Although several case series have been published (*1*,*2*,*9*–*11*), these have been facility-based efforts and, consequently, may not provide an accurate measure of either the incidence or severity of the disease. Such facility-based reports have suggested that deaths from cysticercosis in the United States are uncommon. Although population-based data would provide a more accurate assessment of cysticercosis death rates, such data are scarce. To measure cysticercosis-related death rates in California, we reviewed state mortality records for the 12-year period 1989–2000.

## Methods

### Data Source

Data on deaths were obtained from the State of California, Center for Health Statistics, Office of Vital Records. Completion of a death certificate is required by state law. All death certificates in California require the assignment of a cause or sequence of events leading to death as determined by the attending physician. If a physician is not in attendance, or the death is accidental or occurs under suspicious circumstances, cause of death is determined by the local coroner or medical examiner. Completed death certificates are transmitted from county jurisdictions to the California Department of Health Services, where the causes or sequence of events for each death record are keyed into a computer to create an input data file, which is subsequently sent to the National Center for Health Statistics (NCHS), which produces codes for both the underlying cause of death and multiple cause for each death record. The resulting coded file is then returned to the California Department of Health Services, Office of Vital Records. Data from the State of California’s Multiple Cause of Death Files for the 12-year period 1989–2000 were searched for persons for whom cysticercosis (ICD-9 code 123.1 for years 1989–1998, and ICD-10 code B69 for years 1999 and 2000) was listed as a cause of death. Additional information extracted from the death record included age, gender, race/ethnicity, level of education, country of birth, place of death, and date of death.

### Data Analysis

Cysticercosis death rates per million population were calculated for California and for its major counties by using mid-period population estimates (1994). Population data were obtained from the Department of Finance, Demographic Research Unit, State of California. Crude cysticercosis death rates and 95% confidence intervals were computed according to age, gender, race/ethnicity, and county of residence. Rate ratios and 95% confidence intervals were also calculated. The chi-square, Fisher exact, and Student *t* tests were employed when appropriate to assess apparent differences.

## Results

A total of 124 cysticercosis deaths (mean 10.3 per year) were identified over the 12-year study period, representing a crude 12-year death rate of 3.9 per million population (95% confidence interval [CI] 3.2 to 4.6). Latino residents accounted for 115 (92.7%) of the total deaths recorded, while 5 (4.0%) were white, 3 (2.4%) were Asian, and 1 (0.8%) was black (Table 1). Eighty-two (66.1%) were male; 42 (33.9%) were female. The mean age at death was 39.9 years (range 7–81 years). Most case-patients (107, 86.3%) were born outside the United States, and 90 (72.6%) had emigrated from Mexico. All three fatal cases in Asians were in male immigrants >55 years of age. Cysticercosis was listed as the underlying cause of death for 92 (74.2%) of the patients.

**Table 1:** Demographic characteristics of 124 patients with fatal cysticercosis in California, 1989–2000

Characteristic	No.	%
Sex		
Male	82	66.1
Female	42	33.9
Race/Ethnicity		
White	5	4.0
Black	1	0.8
Latino	115	92.7
Asian/Pacific Islander	3	2.4
Age group (y)		
5–14	3	2.4
15–24	25	20.2
25–34	34	27.4
35–44	16	12.9
45–54	20	16.1
55–64	10	8.1
>65	16	12.9
Educational level (y)		
<12	83	66.9
12	24	19.4
>12	17	13.7
Country of birth		
United States	17	13.7
Mexico	90	72.6
Other	17	13.7

Crude cysticercosis death rates are presented in Table 2. Rates were highest in Latino persons (13.0/10^6^, 95% CI 10.6 to 15.3) compared with those for other racial/ethnic groups (0.4/10^6^, 95% CI 0.1 to 0.7), in males (5.2/10^6^, 95% CI 4.0 to 5.7) relative to females (2.7/10^6^, 95% CI 1.9 to 3.5), and in persons >14 years of age (5.0/10^6^, 95% CI 4.2 to 5.9). Cysticercosis deaths varied by year with the greatest number (16 deaths) observed in 1992 (Figure). The highest cysticercosis death rates were in the counties of Los Angeles (7.7/10^6^), Riverside (6.0/10^6^), and Ventura (5.7/10^6^). More than half of the deaths (70) were among Los Angeles County residents.

**Table 2:** Crude cysticercosis death rates by gender, race/ethnicity, and age group and respective rate ratios, California, 1989–2000

	No.	Rate/10^6^ (95% CI)^a^	RR^b^ (95% CI)
Sex			
Male	82	5.2 (4.0 to 5.7)	1.9 (1.3 to 2.8)
Female	42	2.7 (1.9 to 3.5)	Referent
Race/Ethnicity			
White	5	0.3 (0.04 to 0.5)	Referent
Black	1	0.5 (0 to 1.3)	1.7 (0.2 to 14.3)
Latino	115	13.0 (10.6 to 15.3)	43.3 (17.7 to 106.1)
Asian/Pacific Islander	3	0.9 (0 to 2.0)	3.0 (0.7 to 12.6)
Age group (y)			
5–14	3	0.6 (0 to 1.4)	Referent
15–24	25	5.8 (3.5 to 8.1)	9.7 (2.9 to 32.0)
25–34	34	6.1 (4.1 to 8.2)	10.2 (3.2 to 33.1)
35–44	16	3.1 (1.6, 4.6)	5.2 (1.5 to 17.7)
45–54	20	5.7 (3.2 to 8.3)	9.5 (2.8 to 32.0)
55–64	10	4.4 (1.7 to 7.1)	7.3 (2.0 to 26.6)
>65	16	4.7 (2.4 to 7.1)	7.8 (2.3 to 26.9)

^a^12-year rate calculated by using midpoint (1994) population data. ^b^Rate ratio.

**Figure:**
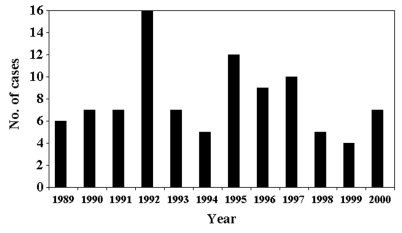
Cysticercosis deaths in California by year, 1989–2000, from state mortality data.

Seventeen cysticercosis deaths occurred in U.S.-born residents, representing 14% of all cysticercosis deaths. U.S.-born patients with fatal cysticercosis had higher educational levels (p = 0.02), were older (mean age 49.1 years versus 38.4 years, p < 0.05), and were more likely to be male (but this difference was not statistically significant) (Table 3). Although 71% of U.S.–born deaths occurred among Latino persons, this figure was lower than the proportion for foreign-born Latino residents (p < 0.01).

**Table 3:** Comparison of selected characteristics of patients born in the United States and those born outside the United States with fatal cysticercosis, California, 1989–2000

	U.S.-born^a^ no (%)	Non–U.S.-born^a^ no. (%)
Sex		
Male	13 (76.5)	69 (64.5)
Female	4 (23.5)	38 (35.5)
Race/Ethnicity		
White	4 (23.5)	1 (0.9)
Latino	12 (70.6)	103 (96.3)
Black	1 (5.9)	0
Asian/Pacific Islander	0	3 (2.8)
Age group		
5–14	0	3 (2.4)
15–24	3 (17.7)	22 (20.6)
25–34	2 (11.8)	32 (29.9)
35–44	3 (17.7)	13 (12.2)
45–54	1 (5.9)	19 (17.8
55–64	2 (11.8)	8 (7.5)
>64	6 (35.3)	10 (9.3)
Educational level (y)		
<12	7 (41.2)	76 (71.0)
12	5 (29.4)	19 (17.8)
>12	5 (29.4)	12 (11.2)

^a^N = 17.

Principal coexisting conditions listed as contributing to death included hydrocephalus in 45 (36.3%) persons and epilepsy or convulsions in 20 (16.1%) deaths. Reported place of death included inpatient facility (69%), emergency room or outpatient clinic (10%), nursing home, (9%) and residence (9%).

## Discussion

Our findings indicate that cysticercosis is an important and preventable cause of premature death in California, particularly among Latino youth, and the disease may be a more common cause of death in the United States than previously recognized. Although fatal cysticercosis principally affects Hispanic immigrants, our findings suggest that this larval tapeworm causes infection and death in U.S.-born residents as well.

Published studies from large facility-based case series have reported that the cysticercosis death rate is relatively low (Table 4). In a review of records from four county hospitals in Los Angeles County, Richards and colleagues identified 497 cysticercosis cases from 1973 to 1983 (*1*). The overwhelming majority of patients were of Latino ethnicity (95%). The observed death rate was 2.2% (11 case-patients). Another study found three (1.3%) fatal cases of cysticercosis among 238 cases identified in a Los Angeles hospital from 1981 to 1986 (*9*). All cases were in Latino patients. A third Los Angeles report involving 230 patients observed two deaths (<1%) in a population composed predominantly of immigrants from Latin America (*10*). A review of neurocysticercosis records from Ben Taub Hospital in Houston, Texas, over the 6-year period 1986–1991 identified 112 cases; 97% were in Latino patients, who were principally from Mexico (*2*). Deaths were uncommon; one patient died. He also had ventricular disease with shunt malfunction complicated by staphylococcal and candidal infections. Forty-seven cases of neurocystcercosis were reported among children whose cases were diagnosed at Children’s Memorial Hospital in Chicago from 1986 to 1994 (*12*). A total of 45 (96%) patients were Latino; however, 19 (42%) were reportedly born in the United States. No deaths were observed in these pediatric case-patients. In a study from Children’s Hospital in Los Angeles covering the period 1980–1986, no deaths occurred among 52 children with neurocysticercosis (*11*). A total of 51 (98.1%) patients were Latino, 29 (57%) of whom were born in the United States. An unweighted estimate of death rates across these studies suggests a case-fatality rate of <1%. However, such facility-based studies, while providing valuable information, have substantial limitations and may have underestimated the impact of cysticercosis as a cause of death. Limited data from a surveillance system established in Los Angeles County from 1988 to 1990 showed a mortality rate of nearly 6% (8 of 138 incident cases); however, this observation was based on small numbers (*6*).

**Table 4:** Hospital-based cysticercosis case series and observed mortality rates, United States

Location	No. cases	No. deaths (%)	Years	Reference
Los Angeles	497	11 (2.2)	1973–1983	1
Los Angeles	238	3 (1.3)	1981–1986	9
Los Angeles	230	2 (0.9)	1981^a^	10
Houston	112	1 (0.9)	1986–1991	2
Chicago	47	0	1986–1994	12
Los Angeles	52	0	1980–1986	11

^a^Range of years not provided.

Both the location of the infection and the number of larvae may have an impact on whether the disease is fatal. Extraparenchymal infection, particularly intraventricular disease with resultant hydrocephalus, has been associated with a poorer prognosis (*13*–*15*). We found that more than one third of patients who died had concurrent hydrocephalus, suggesting intraventricular location of cysts. In addition, ingesting a large number of eggs may cause an overwhelming, fatal acute infection with numerous larvae and notable central nervous system pathology. Cases of racemose cysticercosis, a phenomenon in which cysticerci continue to grow and spread through tissue, may also have a poor prognosis (*16*).

Our data suggest that, although uncommon, cysticercosis-related deaths routinely occur among persons born in the United States, a phenomenon that has not been previously reported. Such deaths in U.S.-born residents may reflect low-level endemic transmission within the United States. Confirmed cysticercosis among persons who have never traveled outside the United States has been repeatedly documented over the past 20 years (*1*,*5*,*6*) and can often be traced to a tapeworm carrier among household members or other close personal contacts. Cysticercosis acquired in the United States may also have been transmitted through consuming imported or local produce contaminated with *T. solium* eggs. *Taenia* eggs can survive for long periods in the environment (*17*), and human feces used as fertilizer or contaminated water employed for irrigation can contaminate crops prior to importation.

Alternatively, cysticercosis among U.S.-born persons may reflect travel-related exposure and infection. Travel by U.S. residents to cysticercosis-endemic areas is common, and exposure to food and water contaminated with the eggs of *T. solium* may readily occur. The recovery of *Taenia* spp. eggs from several varieties of vegetables obtained in local markets in the northeastern Mexican state of Tamaulipas, which borders the United States, has been reported (*18*). Although probable travel-associated cysticercosis has previously been documented (*6*), neither the frequency nor the risk factors for travel-related cysticercosis have been studied.

Nearly one half of those who died of cysticercosis in our study were 15–34 years of age. This represents a heavy toll among persons in young, highly productive age groups. Such a phenomenon is uncommon for most infectious diseases, which typically cause higher rates of death in the very young and the elderly (*19*).

Observed death rates were highest in Los Angeles, Riverside, and Ventura counties. This finding may reflect the fact that residents of these jurisdictions include substantial numbers of immigrants from cysticercosis-endemic areas, particularly Mexico and other areas of Latin America.

Our data, although population-based, likely underestimate cysticercosis deaths for several reasons. Cysticercosis must be recognized and diagnosed for it to be listed on the death certificate. This would require confirmation of infection through biopsy, autopsy or specialized serologic testing (*20*). Consequently, some cases of fatal cysticercosis likely are undiagnosed and unrecognized; this would result in the miscoding of cysticercosis-related deaths as other conditions. For this reason, death records likely possess moderate sensitivity for identification of true cysticercosis deaths. Our findings demonstrate the benefits of using multiple cause of death data instead of the traditional underlying cause of death data alone for estimating the extent of cysticercosis deaths. An additional 32 (26%) cases were identified by using multiple-cause coded files.

Using death certificates to assess the impact of disease has both advantages and limitations. Since submitting a death certificate is required by state law, virtually all deaths are ascertained and registered. The use of death records, therefore, provides population-based data that avoid the potential biases of facility-based or other nonpopulation-based data sources. Death records also provide a good measure of disease trends over time and can be useful in making comparisons between geographic regions. Mortality data can also help indicate disease severity and contribute to measures of disease impact. Limitations of mortality data include the following: inaccurate information due to errors in recording cause of death; coding errors; and misclassification of race/ethnicity (*21*–*23*). Recognizing that deaths from cysticercosis represent only a small fraction of total disease is also important. Finally, both census data and intercensus population estimates used to calculate rates may contain inaccuracies. For these reasons, our estimate of the cysticercosis death rate must be interpreted with caution.

Cysticercosis is a preventable fecal-oral transmitted infection that can cause severe neurologic disease and death and result in substantial cost to the healthcare system. Additional information is needed on the prevalence and incidence of cysticercosis and on cysticercosis-related deaths in the United States. To better define the extent of cysticercosis, state and local health authorities should consider instituting a requirement for the mandatory reporting of this infection. Such surveillance systems should include aggressive efforts to identify possible tapeworm carriers among household members and other close personal contacts. Treating such tapeworm carriers can eliminate sources of infection and prevent additional transmission. Controlled epidemiologic studies to assess risk factors and potential sources for both local and travel-associated cases of cysticercosis should be pursued. Although we could not assess whether problems in access to health care contributed to cysticercosis deaths, nearly 20% of persons with fatal cases died at home, in an emergency room, or in an outpatient setting. Studies to evaluate the possible impact of access issues on cysticercosis deaths would be useful. Collaborative studies with Mexican public health authorities on the prevalence and incidence of cysticercosis in the border regions should be implemented (*24*). While transmission of cysticercosis from a commercial food handler has never been documented such transmission may occur and any food handler with taeniasis (infection with adult *T. solium* or *Taenia* of unknown species) should be precluded from handling food until successfully treated.
